# Microbial communities in marine sediments modify success of an invasive macrophyte

**DOI:** 10.1038/s41598-017-10231-2

**Published:** 2017-08-29

**Authors:** Paul E. Gribben, Shaun Nielsen, Justin R. Seymour, Daniel J. Bradley, Matthew N. West, Torsten Thomas

**Affiliations:** 1 Centre for Marine Bio-Innovation, and School of Biological, Earth and Environmental Sciences, University of New South Wales, Sydney, 2052 Australia; 2 Sydney Institute of Marine Science, 19 Chowder Bay Road, Mosman, NSW 2088 Australia; 3 Plant Functional Biology and Climate Change Cluster, University of Technology, Sydney, 2007 Australia; 4 School of Life Sciences, University of Technology, Sydney, 2007 Australia

## Abstract

Invasive plants have extensive impacts on ecosystem function and biodiversity globally. Our inability to manage invasive species stems in part from a lack of understanding of the processes that control their successful establishment and spread. To date, studies have largely considered how above-ground processes control native/invasive plant interactions. Emerging research from terrestrial and wetland ecosystems demonstrates that below-ground processes under microbial control can determine the outcome of interactions between native and invasive plants. Whether sediment microbes modify the success of invasive macrophytes in marine ecosystems is untested, despite marine sediment microbes controlling many ecological processes (e.g. nutrient cycling) comparable to those in terrestrial ecosystems. We first show that sediment bacterial communities differ between the native seagrass *Zostera capricorni* and the invasive alga *Caulerpa taxifolia* and that those differences relate to functional changes in sulfur cycling between the macrophytes. Second, by experimentally manipulating the microbial communities we show that intact microbial communities in *Z*. *capricorni* sediments provide biotic resistance by reducing *C*. *taxifolia* fragment growth 119% compared to when they are inactive, and intact microbial communities in *C*. *taxifolia* sediments have positive feedbacks by increasing fragment growth 200%. Thus, similar to terrestrial ecosystems, microorganisms appear to indirectly control the success of invasive macrophytes in marine ecosystems.

## Introduction

Understanding the processes driving the establishment and spread of invasive plants is one of the great challenges for managing global biodiversity. Several mechanisms underpinning the establishment of invasive plants have received empirical support (e.g. increased competitive ability, escape from natural enemies and habitat availability; see ref. 1 for review), yet results from these studies are equivocal as they do not adequately explain the successful establishment of many exotic plants^2, 3^ and there is a lack of consensus on which mechanisms are the most important. This problem may, in part, stem from most studies focussing on the direct effects of these mechanisms on above ground processes.

A growing body of evidence from terrestrial and less well studied wetland ecosystems demonstrates that the soil microbiota can control the establishment and spread of invasive plants (see refs 3–5 reviews). Microbial communities have both positive and negative effects on invasion success^6^. For example, native and invasive plants can carry pathogenic organisms that can either inhibit or promote invasion success. Invasion success can also be facilitated by the presence of mutualistic microorganisms (e.g. mycorrhizal fungi, and nitrogen-fixing bacteria)^7, 8^, which enhance the nutrient status of invasive plants potentially altering soil chemistry with negative feedbacks to native plants^9^. Moreover, the outcomes of interactions between native and invasive plants can be altered by removing or inhibiting (e.g. via autoclaving soils) components of the microbial community responsible for controlling interactions between them (e.g. refs 6 and 10). Incorporating belowground microbial processes into our understanding of native/invasive plant interactions may provide a more holistic framework for determining invasive plant success, and may provide context for equivocal tests of other mechanisms.

Despite the developing evidence for the importance of soil microbes in controlling the success of terrestrial invasive plants, whether they modify the success of invasive plants in marine ecosystems is untested. However, there is good reason to suggest they may. Comparable to terrestrial ecosystems, microorganisms in marine sediments exert strong control over ecological processes (e.g. nutrient availability, sediment chemistry) that affect marine macrophytes^11^. Importantly, these processes can differ between interacting native and invasive macrophytes. For example, studies conducted at multiple sites in Australia and the Mediterranean show that sediments colonised by the invasive alga *Caulerpa taxifolia* – one of the 100 most invasive species in the world^12^ – are often hypoxic, and have higher total sulphide pools and higher levels of acid volatile sulphides compared to sediments in native seagrass competitors and unvegetated sediments^13–20^. Chisholm and Moulin^13^ speculated that changes in sediment chemistry are related to a photosynthetic product released from *C*. *taxifolia’s* rhizoids (which embed the alga in sediments) to stimulate a fermenting bacterial community. This bacterial community is hypothesised to provide fermentation products to sulfate reducers resulting in sulphide production. However a detailed analysis of the bacterial community structure and composition in *C*. *taxifolia* sediments has not been performed. Given that *C*. *taxifolia* and native seagrasses will likely release different types and amount of photosynthetic products, their associated sediment should thus harbour different microbial communities, which may differentially affect the success of *C*. *taxifolia*. It has been suggested that increased seagrass canopy may restrict invasion by *C*. *taxifolia* compared to unvegetated areas, and it grows better in unvegetated areas due to an absence of competitors^21^. However, we suggest that invasion of seagrass beds may also be indirectly mediated by sediment microbial communities.

We used 16S rRNA gene sequencing to determine if the structure (and potential function) of bacterial communities differed in sediment beds of *C*. *taxifolia* and the native seagrass *Zostera muelleri* subsp. *capricorni* (Asch.) (hereafter *Z*. *capricorni*). We then tested the hypotheses that the bacterial communities in seagrass and *C*. *taxifolia* inhibit and promote fragment success, respectively. We did this by exposing *C*. *taxifolia* fragments (*C*. *taxifolia* primarily spreads through asexual fragmentation) to sediments from *Z*. *capricorni* and *C*. *taxifolia* that had either intact microbial communities or that were inactivated by autoclaving.

## Results and Discussion

**Figure 1:**
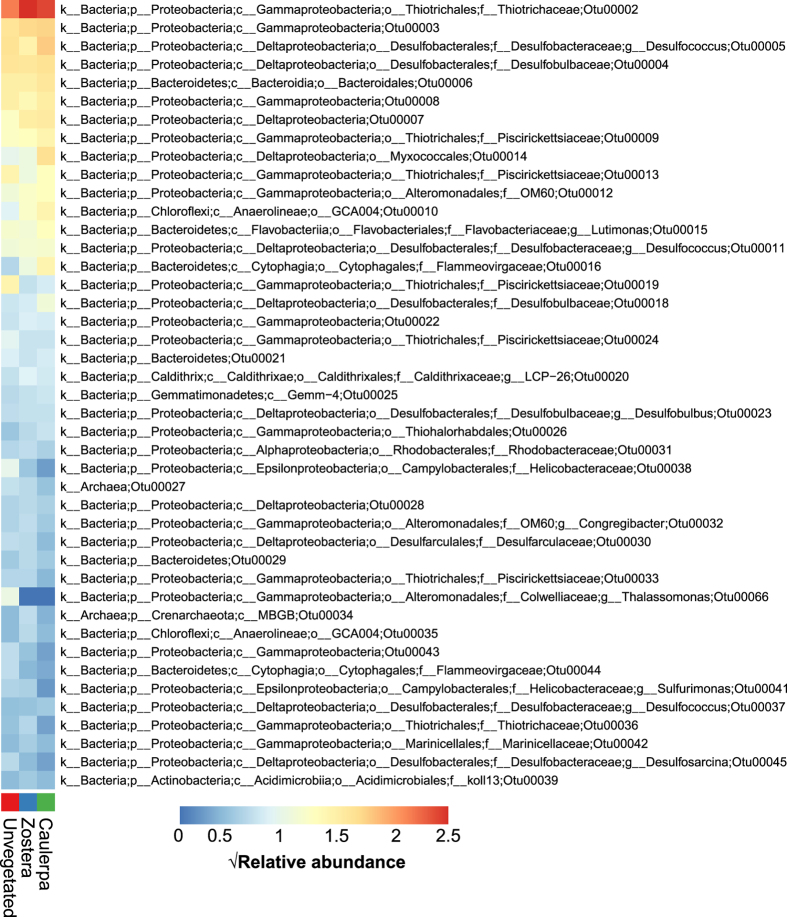
Mean relative abundance (square-root transformed) of the most abundant taxa (n = 43, representing 50% of the total mean relative abundances) among sediments in the absence of macrophytes (unvegetated) or in the presence of *Caulerpa taxifolia* and *Zostera capricorni*.

Bacterial communities from each of three sediment types were diverse, with 3040 ± 29 (mean and SD), 3111 ± 46 and 2968 ± 276 OTUs observed in unvegetated, *C*. *taxifolia* and *Z*. *capricorni* sediments, respectively. Shannon’s diversity indices revealed highest diversity within the unvegetated sediments, and lower diversity within the *C*. *taxifolia* and seagrass sediments, although no significant differences were detected (F_2,6_ = 0.59, *P* = 0.58, Supplementary Fig. S1). Each of the sediment types were generally characterised by the presence of the same bacterial OTUs, but with contrasting abundances. The most abundant OTUs in each of the sediments were associated with the classes Gamma- and Delta-proteobacteria, including the family *Thiotrichaceae* (Gamma-proteobacteria), the families *Desulfobacteraceae* and *Piscirickettsiaceae*, the genus *Desulfococcus*, the order *Myxococcales* (Delta-proteobacteria) and other OTUs only classified to the class level (Fig. 1). There were also abundant OTUs from the order Bacteroidales (phylum Bacteroidetes) and the order GCA004 (phylum Chloroflexi) (Fig. 1). These groups of bacteria are common in estuarine and marine sediments, with functional capacities that cover both aerobic and anaerobic nutrient cycling including nitrogen, sulfur and iron^22, 23^. They have also been identified in the rhizosphere of European seagrasses^24^.

**Figure 2:**
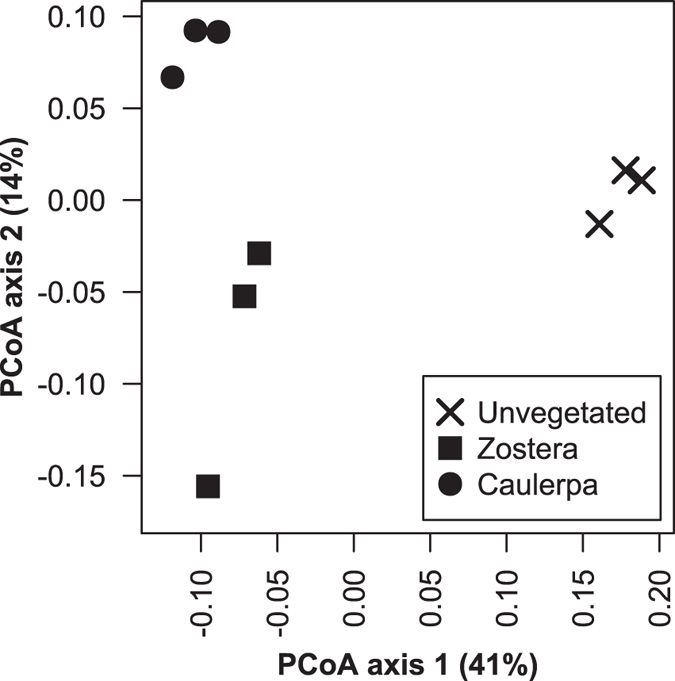
PCoA ordination of Bray-Curtis dissimilarities between bacterial communities from marine sediment in the absence of macrophytes (unvegetated) or in the presence of *Caulerpa taxifolia* and *Zostera capricorni*.

Bacterial communities in sediments associated with macrophytes differed in composition (abundance of OTUs) compared to those of unvegetated sediments (Fig. 2, PERMANOVA contrast: Unvegetated vs. Vegetated, F_1,6_ = 6.75, *P* = 0.002, Supplementary Table 1), and also differed between the two vegetated habitats (PERMANOVA contrast: F_1,6_ = 3.0, *P* = 0.036, Supplementary Table 1 and Fig. 2). Unvegetated sediments shared, on average, 65% overall community similarity with vegetated sediments, while communities within vegetated sediments shared between 72 and 80% similarity. Thus bacterial community composition of sediments was significantly related to the presence/absence of macrophytes in general, and also by macrophyte species.

**Figure 3:**
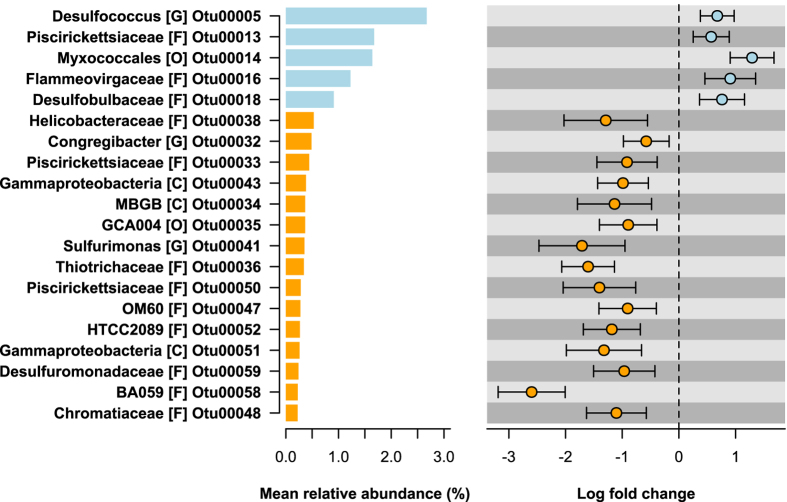
Differentially abundant (DA) OTUs in marine sediment in the presence of *Zostera capricorni* or *Caulerpa taxifolia*. The top 20 DA OTUs chosen by the greatest mean total relative abundances (left column) are shown, along with the log fold change (LFC) in abundance from *Z*. *capricorni* to *C*. *taxifolia* sediments (right column). OTUs are described by their lowest taxonomic assignment ([C] = class, [O] = order, [F] = family, [G] = genus). Mean relative abundanc, LFC and 95% confidence intervals are calculated using negative binomial generalised linear models.

There were 94 OTUs that differed in relative abundance between sediments collected from *C*. *taxifolia* and *Z*. *capricorni* beds (GLM contrast: *P*_adj_ < 0.05, Figs 3, S2 and S3, Supplementary Table 3). These represented 2.4% of the OTUs compared, and which together constituted 34% of the total relative abundance. Sediments occupied by *C*. *taxifolia* had a marked enrichment in OTUs belonging to the phylum Delta-proteobacteria, including the family *Desulfobulbaceae* and the genus *Desulfococcus* (Figs 1, 3 and S3). This family and the genus are generally associated with the reduction of sulfate, sulfite, thiosulfate or sulfur in anaerobic environments^25^ and hence would be driving the production of H_2_S as previously observed in *C*. *taxifolia* sediments by chemical analysis^13, 16, 26^. These bacteria are also chemoorganoheterotrophic with incomplete oxidation of organic matter that often ends in the production of acetate, which could result in acidification of the sediments and explain the lower pH previously observed in *C*. *taxifolia* sediment^16, 17^. In contrast, sediments occupied by *Z*. *capricorni* had greater abundances of members of the Gamma- and Epsilon-proteobacteria, the latter including the genus *Sulfurimonas*. These bacteria have been identified in the oxidation of sulfur in aerobic environments with the production of sulfate^23^. In particular, *Sulfurimonas* is a genus that is strictly chemolithoautotrophic using reduced sulfur compounds (e.g. H_2_S) as electron donors and nitrate, nitrite or oxygen as electron acceptors to produce sulfates^27^. While each sediment type was not devoid of the taxa that were representative of the other sediment type, the changes observed in the bacterial communities associated with *C*. *taxifolia* suggest that these sediments have a reduction in aerobic sulfur cycling and an increase in anaerobic sulfur cycling. Such changes in microbial sulfur cycling could be partially driven by the type and amount of organic material available in the sediments derived from the macrophyte growth. For example, sulfur reduction can be enhanced in sediments containing *C*. *prolifera* compared to the seagrass *Cymodocea nodosa*, with former making greater contribution to the organic materials in sediments than the later^19^. The provision of oxygen to sediments by the seagrass rhizoids may also promote a bacterial community that supports steps of the sulfur cycle (e.g. sulphide oxidation), which does not favour the growth of *C*. *taxifolia* in intact seagrass beds.

**Figure 4:**
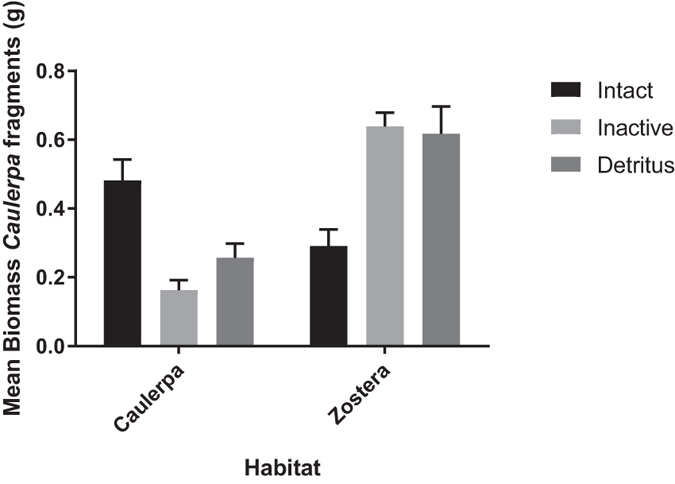
Mean biomass (±SE) of *Caulerpa taxifolia* fragments grown in sediments collected from *C*. *taxifolia* and the seagrass *Zostera capricorni*, in which sediments bacterial communities were intact or inactivate, and in sterile sediments to which *C*. *taxifolia* and *Z*. *capricorni* detritus had been added (n = 12 fragments/treatment combination).

To test whether bacterial communities in seagrass decreased fragment success and microbial communities from *C*. *taxifolia* increased fragment success, we exposed *C*. *taxifolia* fragments to sediments collected from beds of *C*. *taxifolia* and *Z*. *capricorni* that had intact or inactive (via autoclaving) microbial communities. Importantly, the presence or absence of bacterial communities from *C*. *taxifolia* and *Z*. *capricorni* had opposing effects on fragment growth (2-Factor ANOVA: macrophyte x bacterial status interaction, *F*_1,44_ = 53.002, *P* < 0.001); Fig. 4). Our experimental manipulation of sediments demonstrated that intact microbial communities in *Z*. *capricorni* sediments provide some biotic resistance to *C*. *taxifolia* fragments: fragment biomass was 119% higher when the resident microbial community was inactivated (intact vs. inactivated sediments; t_22_ = 4.777, *P* < 0.001; Fig. 4). This result suggests that microbial communities may contribute to why intact seagrass beds remain largely uncolonised by *C*. *taxifolia*. Our experiment further showed that microbial communities in intact *C*. *taxifolia* sediments have positive feedbacks for fragment growth: fragments had 200% higher biomass in the intact microbial community treatment (intact vs. inactivated sediments; t_22_ = −5.549, *P* < 0.001; Fig. 4). Both these processes have previously been demonstrated for terrestrial plants^6^ and here we extend these principles to marine ecosystems.

Because our sediments also contained residual detritus which could have confounded the results, our experiment also contained controls for the potential effects of autoclaving on sediment detritus: we added seagrass detritus and *C*. *taxifolia* detritus to commercially obtained sterile sediments lacking a microbial community. Thus both treatments contained inactive microbial communities but the autoclaved treatments contained autoclaved detrital material whereas the control sediments contained raw detrital material. The fact that there was no difference in fragment growth between microbially inactive sediments and control sediments with raw detritus added for *C*. *taxifolia* (t_22_ = −1.878, *P* < 0.074) or *Z*. *capricorni* (t_22_ = 0.235, *P* = 0.816), suggests any potential effects of autoclaving detritus within the sediment did not influence fragment growth and the results of our experiment can largely be attributed to whether the microbial communities in the sediment were active or not.

Our results may shed light on why dense *Z*. *capricorni* beds appear more resistant to *C*. *taxifolia* invasion than disturbed seagrass beds and unvegetated areas that used to harbour seagrass beds. The established *Z*. *capricorni* beds likely have sediments containing an intricate balance between the products of aerobic (possibly supported by oxygen provisioning by rhizoids to the rhizosphere) and anaerobic sulphur cycling, such as sulfate reduction and H_2_S oxidation, respectively, and this might provide an unfavourable environment for invasion success of *C*. *taxifolia*. However, where seagrass is declining and the aerobic degradation of the detritus is occurring, the environment may become anaerobic because of a lack of oxygenation in the absence of rhizoids and available sulfates will support the metabolic activity of sulfate-reducing bacteria and higher levels of sulphides^13–20^. This transition would not be difficult given the presence of sulfate-reducing bacteria already in seagrass sediments, and in turn would make the environment favourable for the invasion of *C*. *taxifolia*. It has been suggested that invasion of disturbed seagrass beds by *Caulerpa* spp. accelerates their decline because of an increase in sulphides associated with their invasion^18, 19^. Thus while seagrass health declines with increasing sulphide concentrations in the rhizosphere^28, 29^, *C*. *taxifolia* appears relatively tolerant to it, although it clearly needs an active microbial community to do this given the significant decrease in fragment growth in *C*. *taxifolia* sediments once the microbial community was removed.

Our observations suggest an important functional role for microbial communities in *C*. *taxifolia* and *Z*. *capricorni* sediments. Thus, similar to terrestrial ecosystems, microorganisms appear to indirectly modify the establishment and growth of invasive macrophytes in marine soft-sediment ecosystems, providing a unifying framework for understanding invasion success across ecosystems. Importantly, the ability of marine sediments (particularly those occupied by marine macrophytes) to provide biotic resistance to invasion may become eroded through increasing pressure on coastal ecosystems. Moreover, the invasive macroalgae, *C*. *racemosa* and *C*. *taxifolia* may vertically transmit their own microbiota and thus could carry bacteria that would facilitate their invasion into new regions^30, 31^. A full understanding of the importance of microorganisms in mediating the success of invasive species is therefore essential in defining invasion risks to soft-sediment marine ecosystems.

## Methods

### Study Species

*Caulerpa taxifolia* is a coenocytic green alga that has invaded several temperate regions worldwide where it covers large areas of shallow soft-sediment habitat^32, 33^. In New South Wales (NSW), Australia, it was first observed in 2000 and is now present in 13 estuaries^21^. *C*. *taxifolia* forms high-density beds in unvegetated sediments outside seagrass beds^34–36^, has severe impacts on native fauna within the sediments^15, 16, 37–39^ and can outperform native seagrasses^40^. These impacts have been linked to *C*. *taxifolia’s* modification of chemical (increased anoxia and sulphide production) and physical sediment properties^13–17, 26^.

The seagrass *Zostera capricorni* is found from tropical to temperate regions in Australia. It commonly occurs as meadows in mud and sand from 0–7 m depth in estuaries and shallow lagoons. In NSW, *C*. *taxifolia* grows amongst seagrass and is often particularly dense immediately adjacent to seagrass beds^21^.

### Sampling of sediments from Caulerpa taxifolia and Zostera capricorni

Sediment samples were collected from Careel Bay on the mid-eastern coast of Australia (lat/long decimal degrees; −33.6152000, 151.3179000), on 24^th^ April 2015. *C*. *taxifolia* has been present in this region since at least June 2001, where it co-occurs with *Z*. *capricorni*. To determine how sediment microbial communities differed between *C*. *taxifolia* and *Z*. *capricorni*, we collected sediment samples from 100% cover of both macrophytes. We also collected sediment samples from unvegetated areas (0% cover of macrophytes) for comparison. Samples were collected from intermingling patches of unvegetated, *C*. *taxifolia* and *Z*. *capricorni* patches (1 sample/patch; n = 3 patches/treatment) to avoid any potentially spatially confounding effects. All patches of both macrophytes were >5 m^2^. All samples were collected between 1.5 m to 2 m water depth.

As *C*. *taxifolia* is capable of penetrating sediments to a depth of 5 cm via its rhizoids^40, 41^, samples were collected by inserting a core (14.5 mm diameter) at least 1–2 cm into the sediment and, with a gloved thumb over the core opening, removing the core and sediment within. The sediment was slowly released from the core and the top 1 cm collected in 100 mL plastic vials. The vials were immediately placed on ice and transported to the University of New South Wales (UNSW), where they were stored in a −80 °C freezer.

### DNA extraction and 16S rRNA gene sequencing

Total DNA was extracted from 0.5 g of sediment using a PowerSoil DNA extraction kit (MoBio) following manufactures instructions. DNA quality and quantity were checked spectroscopically using a Nanodrop1000 and the bacterial 16S rRNA gene was amplified by PCR using the primers 515F (GTGCCAGCMGCCGCGGTAA) and 806 R (GGACTACHVGGGTWTCTAAT). The V4 regions of this amplicon were sequenced using the MiSeq Reagent Kit v2 (2 × 250 bp) on an Illumina MiSeq platform. Amplification, ligation of sequencing adaptors and sequencing were conducted by the Ramaciotti Centre for Genomics (UNSW, Australia).

### Sequence quality and abundance filtering

Sequences were quality filtered using the software package MOTHUR^42^ following the MiSeq SOP^43^. Briefly, paired sequences were merged into contigs and then aligned in a multiple sequence alignment. Contigs were pre-clustered after which singleton contigs were removed and the resulting contigs were checked for chimeras. Contigs classified as unknown, chloroplast or mitochondria using the Greengenes taxonomic outlines (August 2013 release, ref. 44) with 60% confidence threshold were removed. Operational taxonomic units (OTU) were then formed by clustering contigs at 3% dissimilarity. The total number of OTU counts per sample were equalised using random subsampling. A number of rarefaction curves were generated with sequential removal of rare OTUs to observe the effect of sampling efficiency given removal of rare OTUs (Supplementary Fig. S4). OTUs with <16 total counts were removed, given the position of the rarefaction curve asymptote, which led to a dataset focusing on consistently sampled OTUs. The resulting OTU by sample matrix was used in statistical analysis.

### Effects of macrophyte sediment microbial communities on Caulerpa taxifolia fragment success

We tested the effects of intact and inactive microbial communities from sediments obtained from 100% cover of *C*. *taxifolia* and *Z*. *capricorni* on the success (changes in biomass) of *C*. *taxifolia* fragments. Microbially inactive sediments were produced by autoclaving sediments from both habitats, an approach commonly employed in studies investigating the influence of soil microorganisms on native/invasive plant interactions^6, 9, 45^. To control for the potential effects of autoclaved detritus on sediment chemistry, we added two additional treatments: seagrass or *C*. *taxifolia* detritus added to commercially available sterile sediment (crystalline silica sand; VWR International®). We used a fine white sand product qualitatively similar to the fine grain size and texture of seagrass and *C*. *taxifolia* sediments. Thus both autoclaved sediments and commercial sediments have inactive sediment microbial communities, but still contain detritus from the habitat they were collected.

Thus if no difference in fragment growth between these treatments was observed for both macrophytes, any artefacts of autoclaving on sediment chemistry do not influence fragment growth and any differences in fragment growth between intact and inactivated sediments can be mainly attributed to our manipulation of the microbial communities. All detrital material was ground to a paste prior to addition to aquaria.

To establish the experiment, sediments for the intact and inactive sediment treatments were collected from Careel Bay (as described above) by taking the top 0–2 cm of sediment from the surface only. Sediments were again taken from multiple patches of intermingling of *Z*. *capricorni* and *C*. *taxifolia* (n = 10 patches/macrophyte; patch sizes >5 m^2^). *C*. *taxifolia* fragments used in the experiment were collected from the same patches used to collect *C*. *taxifolia* sediments. In total, l5 litres of sediment from each macrophyte and *C*. *taxifolia* were collected and transported back to the laboratory. On the same day, half the sediment from each macrophyte habitat was autoclaved at 150 °C for 1 hour to create the treatments for the inactive sediment microbial community. The detrital addition treatments were created by adding *Z*. *capricorni* or *C*. *taxifolia* to the commercial sediments. Intact *C*. *taxifolia* or seagrass (above ground biomass only) was collected from a total surface area equivalent to the total surface area (1800 cm^2^) of the aquaria to which it was added. By wet-weight, 0.025 g.cm^2^ and 0.035 g.cm^2^ were added to the *C*. *taxifolia* and *Z*. *capricorni* treatments, respectively. Again, *C*. *taxifolia* and *Z*. *capricorni* were collected from replicate patches at the same time sediment was collected.

Individual sediment treatments were then allocated to replicate 1.1 litre plastic aquaria (15 cm × 10 cm × 10 cm; n = 12 replicates/treatment). Aquaria were filled with 350 ml sediment forming a layer 3 cm deep and sterile autoclaved seawater was added to within 2 cm of the top of the aquaria. For the detrital addition treatments, we added the same amount of commercial sterile sediment to the aquaria and mixed the detrital material through prior to the addition of water. The tanks were then arranged randomly in a temperature-controlled room to maintain the aquaria at a constant 21 °C for the duration of the experiment. This temperature is within the range that maximises the growth of *C*. *taxifolia* fragments^46^. We then created 72 experimental *C*. *taxifolia* fragments each with two fronds, two rhizoids and a five cm long stolon, which were stored in 4 × 20 litre buckets with the same seawater added to the aquaria. Aquaria and fragments were allowed to settle for 24 hours, after which time the biomass of each fragment was determined after gently towel drying the fragments prior to adding them aquaria (n = 1 fragment/aquaria). Each fragment was gently attached to the sediment surface using two plastic U-shaped pins. There was no difference in the average (±SE) fragment biomass among the six sediment treatments at the beginning of the experiment (1-way ANOVA, *F*_5,66_ = 1.323, *P* = 0.265). Half the water in the aquaria was carefully exchanged every second day with sterile autoclaved water preconditioned to 21 °C. The experiment was maintained under fluorescent lightly using a 12:12 hr light/dark cycle. After 5 weeks, individual fragments were removed from the aquaria, towel dried and reweighed.

### Statistical analyses

A one-way design was used to examine differences in sediment microbial communities between unvegetated areas, and in the presence of *C*. *taxifolia* or *Z*. *capricorni*. Specific comparisons were made by use of contrasts, including unvegetated sediment vs. both macrophytes and *C*. *taxifolia* vs. *Z*. *capricorni*.

The Shannon’s diversity index was used to compare alpha diversity among samples. Community-level comparisons (Beta-diversity) were conducted using distance-based methods. OTU counts were log +1 transformed and the Bray-Curtis dissimilarity coefficient calculated between each and every sample pair. The resulting distance matrix was visualised using principle coordinate analysis (PCoA). PERMANOVA was used to examine the effect of sediment source on Bray-Curtis dissimilarities using 999 permutations^47^, and contrasts conducted therein. Distance-based analyses were conducted using the R package ‘vegan’^48^.

Generalised linear models (GLMs) were used to examine which OTUs differed between sediment communities, and we only focused here on the contrast between *C*. *taxifolia* and *Z*. *capricorni*. Counts were modelled using a negative binominal distribution given a strong mean-variance relationship. *P-*values were adjusted for multiple comparisons, and OTUs with *P*_adj_ < 0.05 considered as differentially abundant (DA). OTU comparisons were conducted using the R package ‘DESeq. 2’^49^.

*C*. *taxifolia* fragment success (biomass) was examined using a two-way design including Sediment origin (fixed factor, two levels – *Z*. *capricorni* and *C*. *taxifolia*) crossed with microbial community status (fixed factor, two levels - intact and inactivated) using Analysis of Variance (ANOVA). Independent sample t-tests were used to determine differences in fragment growth between microbial community status within sediment origin and between *Z*. *capricorni* and *C*. *taxifolia* within microbial community status, with Bonferroni corrected alpha values used (α = 0.0125). To test for the potential effects of autoclaving sediments on fragment success, we used t*-*tests to compare *C*. *taxifolia* fragment growth between sterile sediments + *C*. *taxifolia* detritus and autoclaved *C*. *taxifolia* sediments, as well as commercial sterile sediments + *Z*. *capricorni* detritus and autoclaved *Z*. *capricorni* sediments. All fragment analyses were conducted in SPSS v.23.

## Electronic supplementary material

Supplementary Information

Supplementary Table 2

Supplementary Table 3

## References

[1] Levine JM (2003). Mechanisms underlying the impacts of exotic plant invasions. Proc. R. Soc. Lond..

[2] Klironomos JN (2002). Feedback with soil biota contributes to plant rarity and invasiveness in communities. Nature.

[3] van der Putten WH, Klironomos JN, Wardle DA (2007). Microbial ecology of biological invasions. Isme Journal.

[4] Cipollini D, Rigsby CM, Barto EK (2012). Microbes as Targets and Mediators of Allelopathy in Plants. J. Chem. Ecol..

[5] Kowalski, K. P. *et al*. Advancing the science of microbial symbiosis to support invasive species management: a case study on Phragmites in the Great Lakes. *Frontiers in Microbiology* **6**, doi:10.3389/fmicb.2015.00095 (2015). 10.3389/fmicb.2015.00095 PMC4333861 25745417

[6] Callaway RM, Thelen GC, Rodriguez A, Holben WE (2004). Soil biota and exotic plant invasion. Nature.

[7] Ernst M, Mendgen KW, Wirsel SGR (2003). Endophytic Fungal Mutualists: Seed-Borne Stagonospora Spp. Enhance Reed Biomass Production in Axenic Microcosms. Molecular Plant-Microbe Interactions.

[8] Reed MLE, Warner BG, Glick BR (2005). Plant Growth–Promoting Bacteria Facilitate the Growth of the Common Reed Phragmites australisin the Presence of Copper or Polycyclic Aromatic Hydrocarbons. Current Microbiology.

[9] Reinhart KO, Callaway RM (2006). Soil biota and invasive plants. New Phytologist.

[10] Mangla S, Callaway RM (2008). Exotic invasive plant accumulates native soil pathogens which inhibit native plants. J. Ecol..

[11] Marinelli, R. L. & Waldbasser, G. G. In *Interactions between macro- and microorganisms in marine sediments* (eds E. Kristensen, R.H., Hease, & J. E., Kostka) 233–249 (American Geophysical Union, 2005).

[12] 100 of the world’s worst invasive alien species: A selection from the global invasive species database (The IUCN Invasive Species Specialist Group (ISSG). 12pp 2000).

[13] Chisholm JRM, Moulin P (2003). Stimulation of nitrogen fixation in refractory organic sediments by *Caulerpa taxifolia* (Chlorophyta). Limnol. Oceanogr..

[14] Gallucci, F., Hutchings, P., Gribben, P. & Fonseca, G. Habitat alteration and community-level effects of an invasive ecosystem engineer: a case study along the coast of NSW, Australia. *Mar*. *Ecol*. *Prog*. *Ser*. **449** (2012).

[15] Gribben PE, Byers JE, Wright JT, Glasby TM (2013). Positive versus negative effects of an invasive ecosystem engineer on different community components. Oikos.

[16] Gribben PE (2009). Reduced performance of native infauna following recruitment to a habitat-forming invasive marine alga. Oecologia.

[17] McKinnon JG, Gribben PE, Davis AR, Jolley DF, Wright JT (2009). Differences in soft-sediment macrobenthic assemblages invaded by Caulerpa taxifolia compared to uninvaded habitats. Mar. Ecol. Prog. Ser..

[18] Garcias-Bonet N, Marbà N, Holmer M, Duarte CM (2008). Effects of sediment sulfides on seagrass Posidonia oceanica meristematic activity. Mar. Ecol. Prog. Ser..

[19] Holmer M, Duarte CM, Boschker HTS, Barrón C (2004). Carbon cycling and bacterial carbon sources in pristine and impacted Mediterranean seagrass sediments. Aquatic Microbial Ecology.

[20] Holmer M, Marbà N, Lamote M, Duarte C (2009). Deterioration of sediment quality in seagrass meadows (*Posidonia oceanica*) invaded by macroalgae *(Caulerpa s*p.). Estuaries Coasts.

[21] Glasby TM (2013). Caulerpa taxifolia in seagrass meadows: killer or opportunistic weed?. Biol. Inv..

[22] Baker BJ, Lazar CS, Teske AP, Dick GJ (2015). Genomic resolution of linkages in carbon, nitrogen, and sulfur cycling among widespread estuary sediment bacteria. Microbiome.

[23] Pjevac P, Kamyshny A, Dyksma S, Mussmann M (2014). Microbial consumption of zero-valence sulfur in marine benthic habitats. Environ Microbiol.

[24] Cúcio, C., Engelen, A. H., Costa, R. & Muyzer, G. Rhizosphere Microbiomes of European Seagrasses Are Selected by the Plant, But Are Not Species Specific. *Frontiers in Microbiology* **7**, doi:10.3389/fmicb.2016.00440 (2016). 10.3389/fmicb.2016.00440 PMC4815253 27065991

[25] Kuever, J. In *The Prokaryotes: Deltaproteobacteria and Epsilonproteobacteria* (eds Eugene Rosenberg *et al*.) 75–86 (Springer Berlin Heidelberg, 2014).

[26] Eyre BD, Maher D, Oakes JM, Erler DV, Glasby TM (2011). Differences in benthic metabolism, nutrient fluxes, and denitrification in Caulerpa taxifolia communities compared to uninvaded bare sediment and seagrass (Zostera capricorni) habitats. Limnol. Oceanogr..

[27] Takai K (2006). Sulfurimonas paralvinellae sp. nov., a novel mesophilic, hydrogen- and sulfur-oxidizing chemolithoautotroph within the Epsilonproteobacteria isolated from a deep-sea hydrothermal vent polychaete nest, reclassification of Thiomicrospira denitrificans as Sulfurimonas denitrificans comb. nov. and emended description of the genus Sulfurimonas. International Journal of Systematic and Evolutionary Microbiology.

[28] Terrados J (1999). Are seagrass growth and survival constrained by the reducing conditions of the sediment?. Aquat. Bot..

[29] Duarte, C. M., Holmer, M. & Marba, N. In *Interactions Between Macro-and Microorganisms in Marine Sediments* (eds E. Kristensen, J. E., Kostka & R. H., Haese) 31–60 (Coastal and Estuarine Studies, American Geophysical Union, 2005).

[30] Aires T, Serrão EA, Kendrick G, Duarte CM, Arnaud-Haond S (2013). Invasion is a community affair: Clandestine followers in the bacterial community associated to green algae, *Caulerpa racemosa*, track the invasion source. PLoS ONE.

[31] Arnaud-Haond, S. *et al*. Entangled fates of holobiont genomes during invasion: nested bacterial and host diversities in Caulerpa taxifolia. *Molecular Ecology*, n/a-n/a, doi:10.1111/mec.14030 (2017). 10.1111/mec.14030 28133884

[32] Creese RG, Davis AR, Glasby TM (2004). Eradicating and preventing the spread of the invasive alga *Caulerpa taxifolia* in NSW. NSW Fisheries Final Report Ser.

[33] Meinesz A (2001). The introduced marine alga *Caulerpa taxifolia* in the Mediterranean. Biol. Inv..

[34] Meinesz A (1995). The introduced green alga *Caulerpa taxifolia* in the Mediterranean sea. Bot. Mar..

[35] Wright JT (2005). Differences between native and invasive *Caulerpa taxifolia:* a link between asexual fragmentation and abundance in invasive populations. Mar. Biol..

[36] Wright JT, Davis AR (2006). Demographic feedback between clonal growth and fragmentation in an invasive seaweed. Ecology.

[37] Gribben PE, Wright JT (2006). Sublethal effects on reproduction in native fauna: are females more vulnerable to biological invasion?. Oecologia.

[38] Wright JT, Byers JE, Koukoumaftsis LP, Ralph PJ, Gribben PE (2010). Native species behaviour mitigates the impact of habitat-forming invasive seaweed. Oecologia.

[39] Wright JT, Gribben PE, Byers JE, Monro K (2012). Invasive ecosystem engineer selects for different phenotypes of an associated native species. Ecology.

[40] Ceccherelli G, Cinelli F (1997). Short-term effects of nutrient enrichment of the sediment and interactions between the seagrass *Cymedocea nodosa* and the introduced green alga *Caulerpa taxifolia* in a Mediterranean bay. J. Exp. Mar. Biol. Ecol..

[41] Ceccherelli G, Sechi N (2002). Nutrient availability in the sediment and the reciprocal effects between the native seagrass *Cymodocea nodosa* and the introduced rhizophytic alga *Caulerpa taxifolia*. Hydrobiologia.

[42] Schloss PD (2009). Introducing mothur: open-source, platform-independent, community-supported software for describing and comparing microbial communities. Appl. Environ. Microbiol..

[43] Kozich JJ, Westcott SL, Baxter NT, Highlander SK, Schloss PD (2013). Development of a dual-index sequencing strategy and curation pipeline for analyzing amplicon sequence data on the MiSeq Illumina sequencing platform. Applied and environmental microbiology.

[44] DeSantis TZ (2006). Greengenes, a chimera-checked 16S rRNA gene database and workbench compatible with ARB. Applied and environmental microbiology.

[45] Stinson KA (2006). Invasive Plant Suppresses the Growth of Native Tree Seedlings by Disrupting Belowground Mutualisms. PLoS Biol..

[46] Komatsu T, Meinesz A, Buckles D (1997). Temperature and light responses of alga Caulerpa taxifolia introduced into the Mediterranean Sea. Mar. Ecol. Prog. Ser..

[47] Anderson MJ (2001). A new method for non-parametric multivariate analysis of variance. Austral Ecol..

[48] vegan: Community Ecology Package v. R package version 2.3–4 (2016).

[49] Love MI, Huber W, Anders S (2014). Moderated estimation of fold change and dispersion for RNA-seq data with DESeq. 2. Genome biology.

