# Supplementary material for: Microbial communities in marine sediments modify success of an invasive macrophyte
Source: Sci Rep. 2017 Aug 29;7:9845. doi: 10.1038/s41598-017-10231-2 (PMC5575248; doi:10.1038/s41598-017-10231-2)
Supplement: Supplementary file 1 — Supplementary Information [file 41598_2017_10231_MOESM1_ESM.pdf]

Microbial communities in marine sediments control success of an invasive macrophyte

Paul E. Gribben, Shaun Nielsen, Justin R. Seymour, Daniel J. Bradley, Matthew N. West and Torsten Thomas

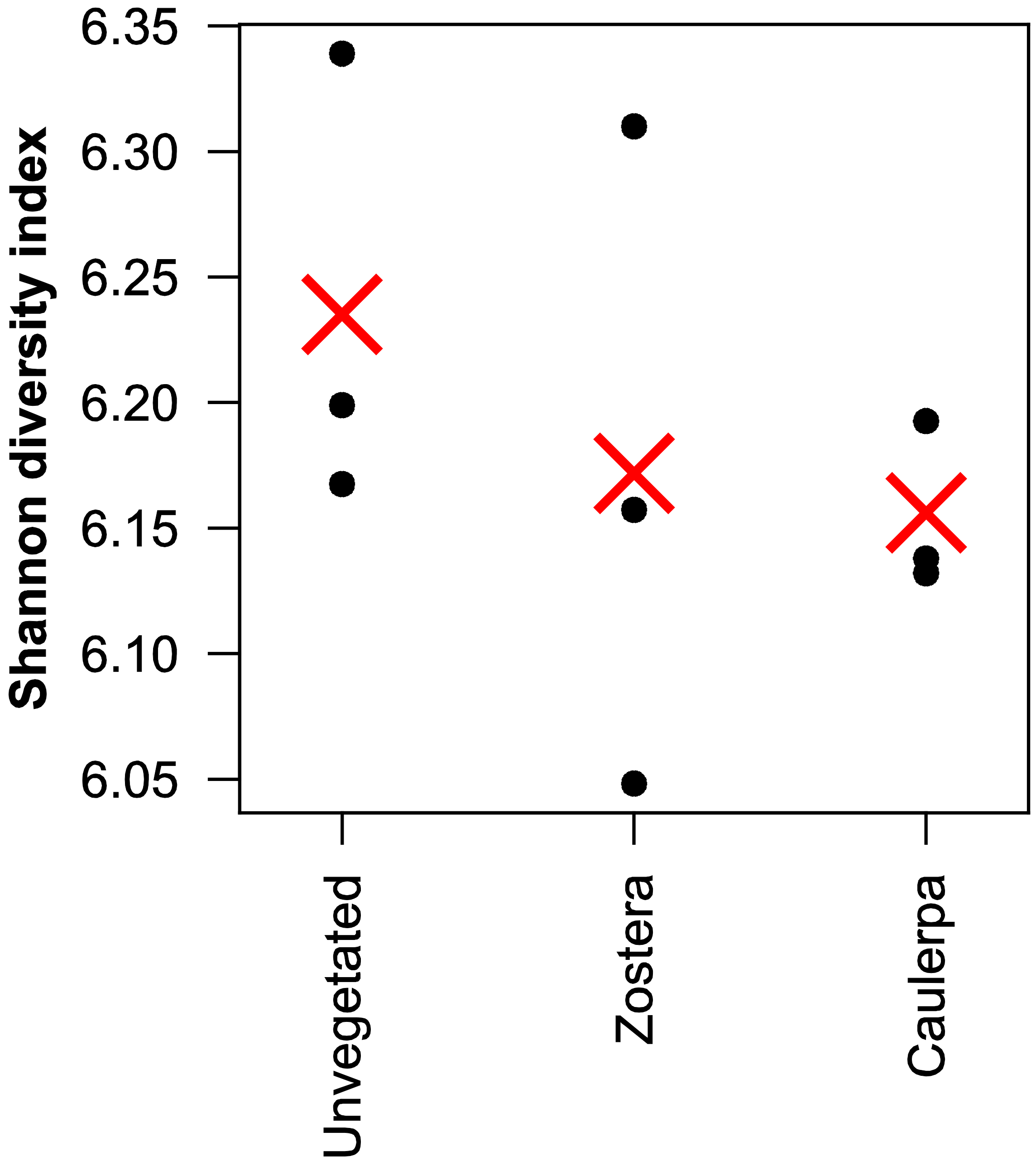

Supplementary Figure S1. Mean (red cross) and sample (black circle) Shannon's diversity indices of microbial communities in marine sediment taken in the absence of macrophytes (Unvegetated) or in the presence of *Caulerpa taxifolia* (Caulerpa) and *Zostera capricorni* (Zostera).

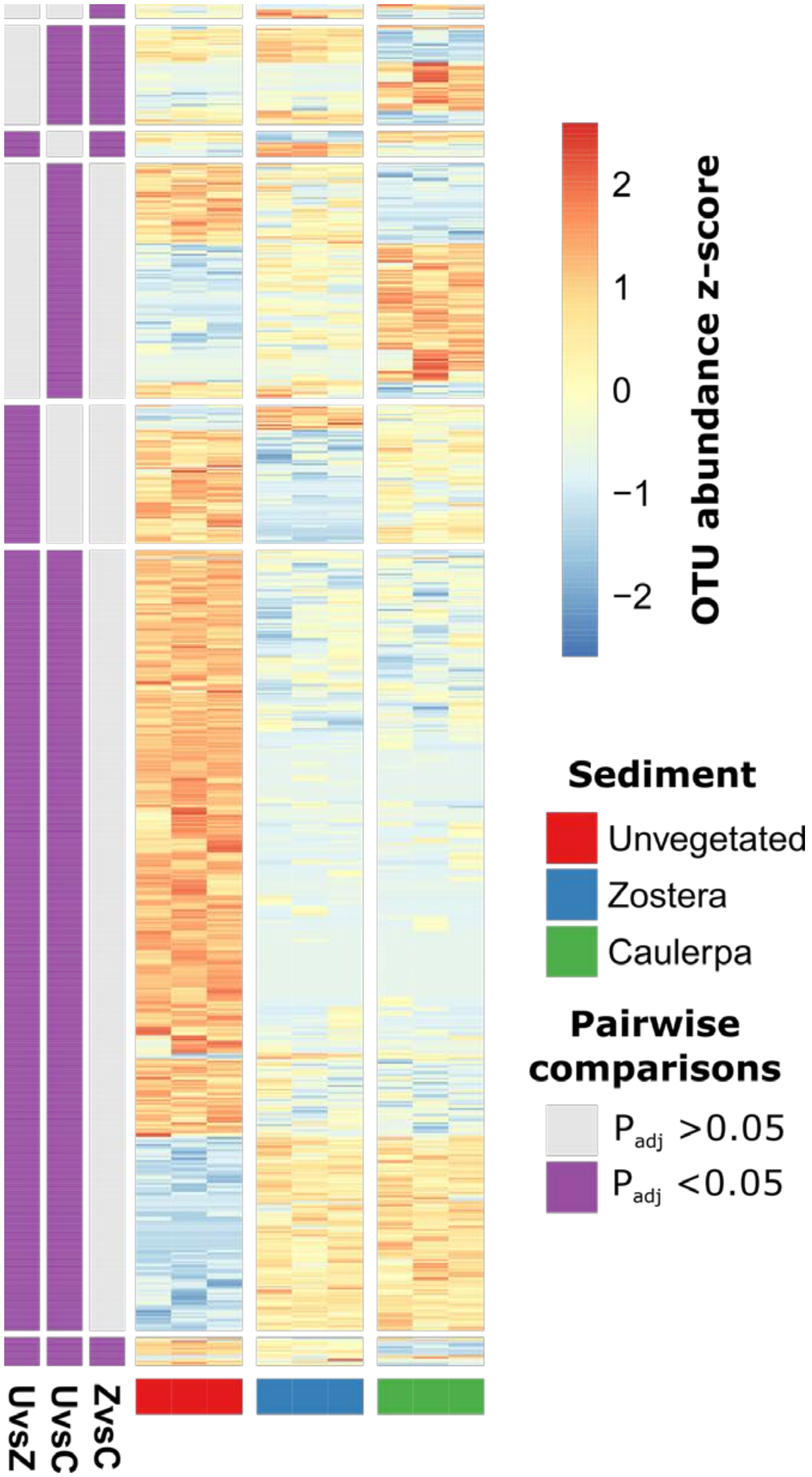

Supplementary Figure S2: Differentially abundant OTUs ($P_{adjusted}$ < 0.05) between marine sediment taken in the absence of macrophytes (unvegetated, U) or in the presence of *Caulerpa taxifolia* (C) and *Zostera capricorni* (Z). OTU abundances have been standardised (z-scores) and ordered according to pair-wise groupings (purple and grey bars on the left of the heatmap). Full details of taxonomic names presented in the heatmap are described in Supplementary Tables 1 and 2.

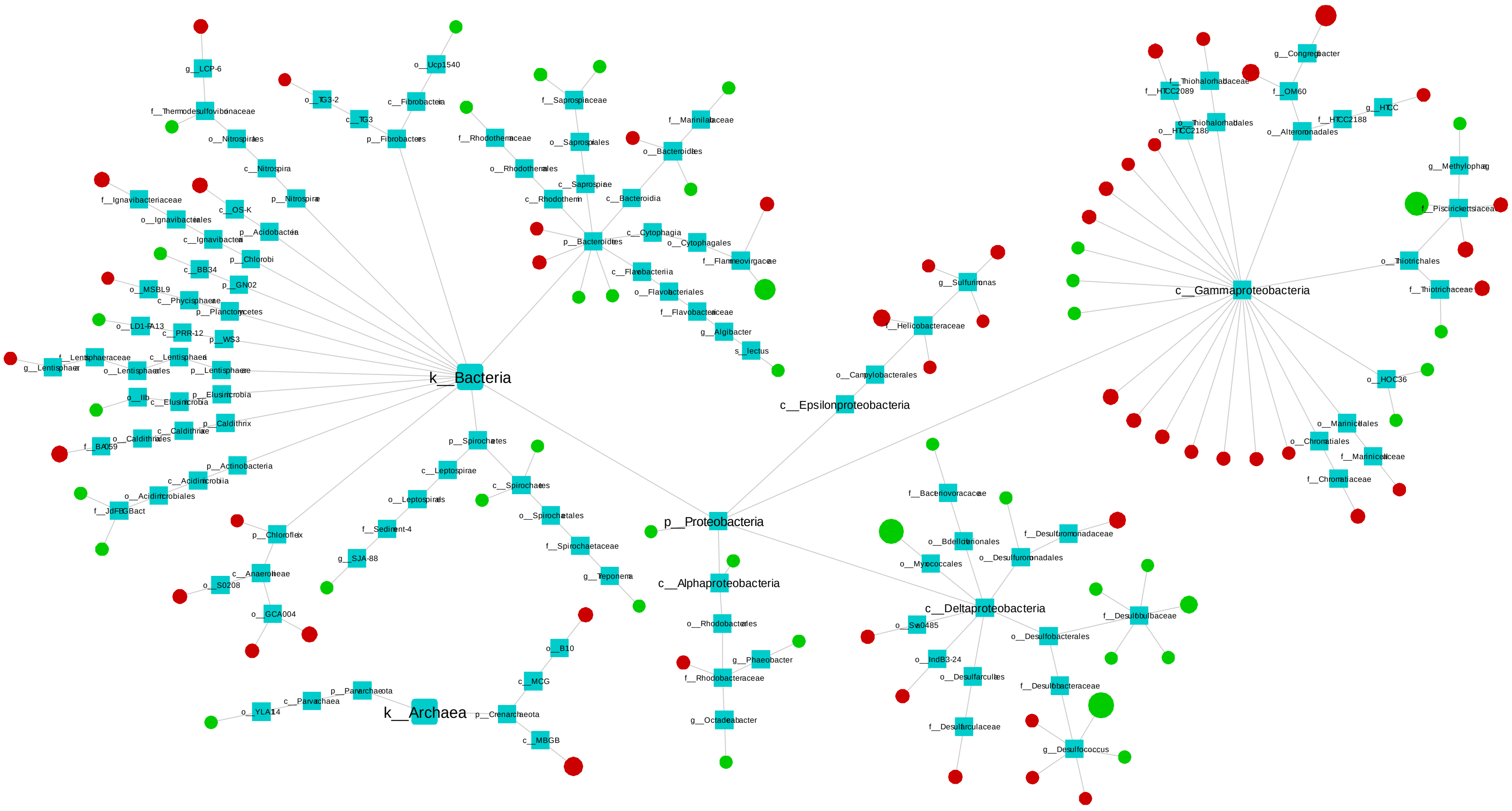

Supplementary Figure S3. Taxonomic rank network of OTUs that were differentially abundant ($P_{adjusted} < 0.05$) in marine sediment in the presence of *Caulerpa taxifolia* (C) or *Zostera capricorni* (Z). Terminal circular nodes represent OTUs, are coloured green or red to represent greater abundances in *C. taxifolia* or *Z. Capricorni*, respectively, and have sizes proportional to relative abundances.

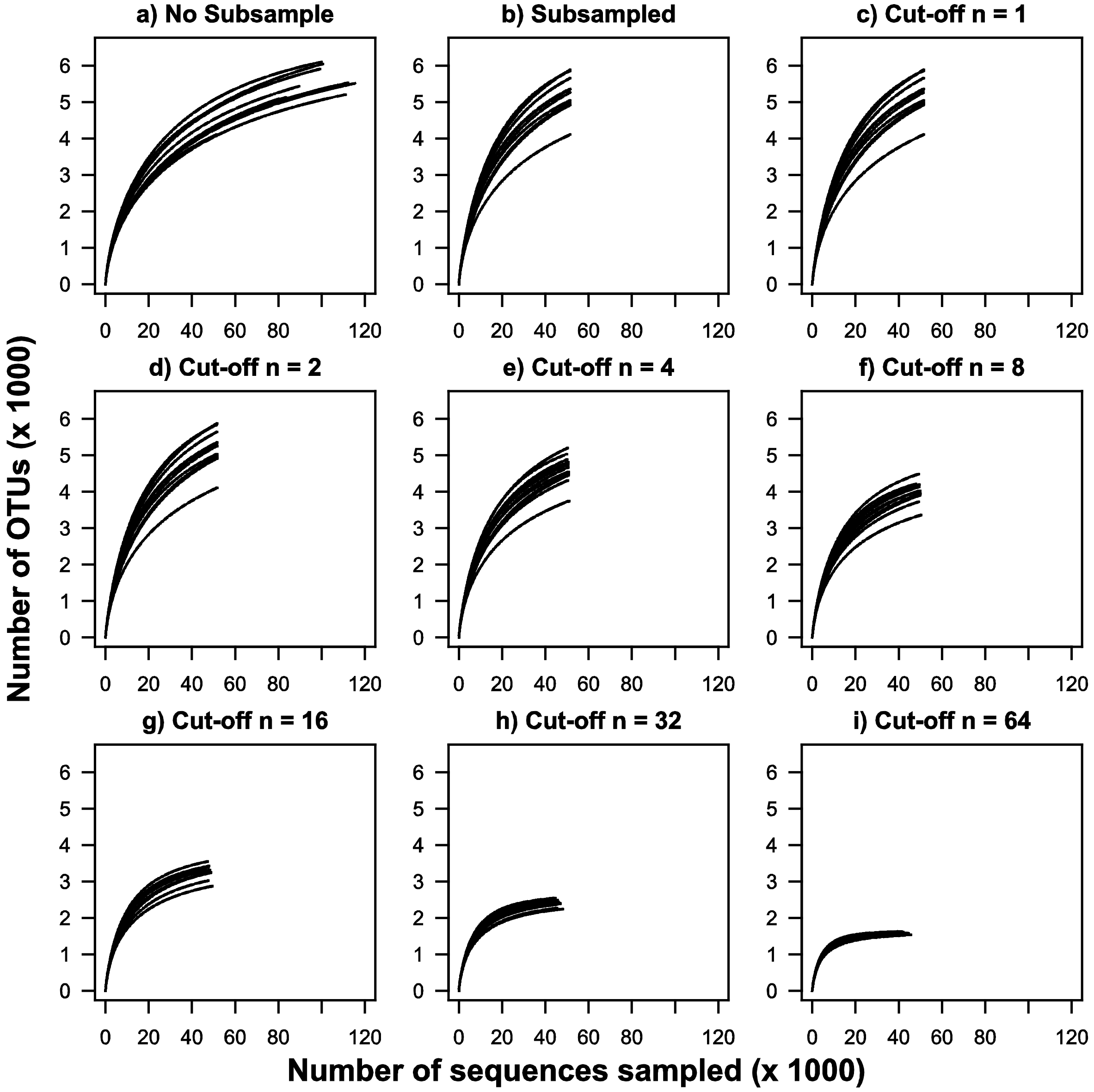

Supplementary Figure S4. Rarefaction curves of bacterial communities a) prior to random subsampling, b) after subsampling each sample to a total of 51432 counts and c-i) with sequential removal of rare OTUs below a defined total count cut-off (i.e. the sum of counts across all samples).

Supplementary Table 1. PERMANOVA of Bray-Curtis similarities among marine sediments in the absence of macrophytes (U) or in presence of *Caulerpa taxifolia* (C) or *Zostera muelleri* (Z). P values were generated using 999 permutations. Contrasts between sediments italised.

**Source**	**Df**	**SS**	**MS**	**F**	**P**
All sediments	2	0.217	0.108	4.876	0.004
U vs ZC	1	0.150	0.150	6.754	0.002
Z vs C	1	0.067	0.067	2.997	0.036
Residuals	6	0.133	0.022		
Total	8	0.350			
